# PLXND1-mediated calcium dyshomeostasis impairs endocardial endothelial autophagy in atrial fibrillation

**DOI:** 10.3389/fphys.2022.960480

**Published:** 2022-08-09

**Authors:** Mengjia Sun, Zhen Chen, Yuanbin Song, Bo Zhang, Jie Yang, Hu Tan

**Affiliations:** ^1^ Institute of Cardiovascular Diseases of PLA, the Second Affiliated Hospital, Army Medical University (Third Military Medical University), Chongqing, China; ^2^ Department of Cardiology, the Second Affiliated Hospital, Army Medical University (Third Military Medical University), Chongqing, China

**Keywords:** atrial fibrillation, PLXND1, endocardial endothelial cell, autophagy, calcium flux

## Abstract

Left atrial appendage (LAA) thrombus detachment resulting in intracranial embolism is a major complication of atrial fibrillation (AF). Endocardial endothelial cell (EEC) injury leads to thrombosis, whereas autophagy protects against EEC dysfunction. However, the role and underlying mechanisms of autophagy in EECs during AF have not been elucidated. In this study, we isolated EECs from AF model mice and observed reduced autophagic flux and intracellular calcium concentrations in EECs from AF mice. In addition, we detected an increased expression of the mechanosensitive protein PLXND1 in the cytomembranes of EECs. PLXND1 served as a scaffold protein to bind with ORAI1 and further decreased ORAI1-mediated calcium influx. The decrease in the calcium influx-mediated phosphorylation of CAMK2 is associated with the inhibition of autophagy, which results in EEC dysfunction in AF. Our study demonstrated that the change in PLXND1 expression contributes to intracellular calcium dyshomeostasis, which inhibits autophagy flux and results in EEC dysfunction in AF. This study provides a potential intervention target for EEC dysfunction to prevent and treat intracardiac thrombosis in AF and its complications.

## Introduction

Atrial fibrillation (AF) is the most prevalent cardiac arrhythmia and affects over 30 million patients worldwide (Virani et al., 2020). It is characterized by rapid and disorganized electrical activity within the cardiac atria. The burden of AF has continued to rise globally, with no signs of waning. The most serious and common complication of AF is stroke, which is caused by the shedding of the intracardiac thrombus (Andrade et al., 2014).

It is generally believed that vascular endothelial cell injury, blood hypercoagulability, and stasis are the main causes of thrombosis (Watson et al., 2009). In comparison to sinus rhythm, AF is characterized by an irregular heart rhythm and loss of effective contraction of the atria. Due to the loss of effective contraction and the special anatomical structure of the left atrial appendage (LAA), blood flow is slow and turbulence occurs in AF condition (Schnieder et al., 2019; Safavi-Naeini and Rasekh, 2020). This leads to blood flow stagnation and susceptibility to thrombosis in the atria, which flows with blood to the brain and results in intracranial embolisms (Fatkin et al., 1994). The endocardial endothelium is a thin structure that directly experiences abnormal blood flow in the atrium (Brutsaert et al., 1996). Therefore, the dysfunction of endocardial endothelial cells (EECs) is considered to be a major cause of thrombosis (Wu and Thiagarajan, 1996). The maintenance of EEC homeostasis in an abnormal blood flow microenvironment seems to be particularly important for endocardial protection and thrombosis inhibition.

Autophagy is the process of maintaining cellular homeostasis. It occurs in all eukaryotic cells and involves the sequestration of cytoplasmic components in double-membrane autophagosomes. Research has indicated the importance of autophagy in regulating endothelial cell (EC) function (Bravo-San Pedro et al., 2017; Mameli et al., 2021). With the development of single-cell analyses, ECs can be reassigned to arterial ECs, venous ECs, lymphatic ECs, and EECs (Tucker et al., 2020). Single-cell analysis results have demonstrated significant differences among these subclusters of ECs, suggesting that studies should focus on a single type of ECs. To date, little information is available on EECs, particularly in AF. Therefore, we established an AF mouse model and isolated EECs. We speculated that the abnormal blood flow shearing force in the atrium transforms mechanical changes into biological signals through baroreceptors, which inhibit autophagy and impair EECs function. These results might provide a novel perspective on the inhibition of thrombosis in atria and stroke prevention in AF progression.

## Materials and methods

### Development of atrial fibrillation mouse models

This study was approved by the laboratory Animal Welfare and Ethics Committee of Third Military Medical University (No. AMUWEC20210494) and all procedures conformed to the standards set by the Declaration of Helsinki. Since homozygous null mice are growth-arrested and die by severe heart defects, heterozygote mice of both alleles (*Tbx5* ^*fl/+*^; CMV-Cre) was established using the CRISPR/Cas9 system at Cyagen. Briefly, a mixture of Cas9 protein (M0646M, NEB), sgRNA, and the donor vector containing loxP sites was injected into fertilized mouse eggs. *Tbx5* ^*fl/fl*^ mice were mated with CMV-Cre transgenic mice (Cyagen Biosciences) to constitutively delete the sequences between loxP sites (KO region: ∼3,240 bp). Tbx5 heterozygous null, wild-type, and floxed mice were genotyped by tail genomic PCR. Age matched CMV-Cre mice were used as SR control group. The duration of AF was monitored using an electrocardiogram (*n* = 6/group).

### Histological analysis

Mice were anesthetized with pentobarbital (50 mg/kg). The hearts from sinus rhythm (SR) and AF mice (*n* = 6/group) were quickly removed through a thoracotomy and rinsed, and then were placed in 4% paraformaldehyde, dehydrated in graded concentrations of ethanol, and embedded in paraffin. Sections (5 μm thick) were cut on a microtome with a disposable blade and stained with hematoxylin–eosin and Masson’s trichrome stain. The cardiomyocyte cross-sectional area (CSA) was analyzed by staining the heart sections with wheat germ agglutinin–Alexa Fluor^®^ 647 conjugate (W32466, Invitrogen). Six mice from each group were subjected to the histological analysis. A minimum of five cross-sections of each heart were examined, and the measurements were averaged for statistical analysis. ImageJ software (RRID:SCR_003070) was used to quantify all histological endpoints.

### Enzyme-linked immunosorbent assay

Detection kits for mouse plasma B-type natriuretic peptide (BNP), troponin I (TnI), and creatine kinase MB (CK-MB) were purchased from Jiangsu Jingmei Biological Technology Co. Ltd. (Jiangsu, China). Approximately 1.5 ml orbital vein blood was drawn from each mouse and stored in procoagulant tubes. Plasma was separated by centrifugation (3,000 x g, 20 min) after coagulation at room temperature for 10 min. The plasma levels of BNP, TnI, and CK-MB were measured using commercially available BNP enzyme-linked immunosorbent assay (ELISA) kits (JM-02343M2, 210727B8), CK-MB ELISA kits (JM-03084M2, 210727C6), and TnI ELISA kits (JM-02662M2, 210727I4) according to the manufacturer’s instructions.

### Isolation of primary mouse endocardial endothelial cell

Primary EECs were isolated from mice using a modified method (Klein et al., 2020). Briefly, the mice were euthanized with CO_2_, and the chests were carefully opened to obtain the hearts. This was followed by three washes with 50 ml of cold PBS to remove excess blood. The left and right atria were carefully cut without contamination from the outer surface and minced into 1 mm^3^ pieces under a microdissection microscope. Atrium tissues containing the endocardium were placed in a separate 5-ml tube containing 1 ml of the digestion buffer for 10 min at 37°C, followed by the addition of 4 ml of EC medium to terminate the digestion. The tubes were then centrifuged at 300 × g for 10 min at room temperature and resuspended in 2 ml of red blood cell lysis buffer (00-4333-57, eBioscience). The pellet was resuspended in EC medium (containing 10% FBS, 50 U/mL penicillin/streptomycin, and 1% L-glutamine) and plated on a six-well plate with the number of seeded wells corresponding to the number of processed hearts. After 24 h incubation in a 5% CO_2_ incubator at 37°C, the medium was changed every 2 days to maintain the EEC cultures and cells were cultured for 4–6 days. EEC clusters were used for experiments described below.

### Purification of endocardial endothelial cell by fluorescence-activated cell sorting.

Fresh cells containing EECs were centrifuged and resuspended in FACS buffer. The antibodies used for the identifying EECs were similar as previously described (Klein et al., 2020). Next, the collected cells were co-stained with a mixture of anti-NPR3 AF647 antibody (NBP2-72881 AF647, Novus Bio; 1:200) and anti-CDH11 AF488 antibody (FAB17901G, R&D Systems; 1:200). After staining with the listed markers for 45 min in the dark at 4°C, samples were washed twice with PBS and transferred to a cell strainer to obtain a single-cell suspension. Cells were resuspended in FACS buffer containing 25 μg/ml DAPI (Sigma-Aldrich) prior to flow cytometry analysis. Cells were first gated on DAPI to exclude dead cells then sorted as NPR3-AF647 and CDH11-AF488 double-positive cells using a flow cytometer (Beckman Gallios). Flow cytometry data were acquired using a FACS Canto flow cytometer with FACS Diva software (BD Biosciences) and analyzed using FlowJo software (v10.7.1). Finally, the pellet of the labeled cell population was resuspended in ECM containing 20% fetal bovine serum (FBS 10102, TransSerum) and incubated in a 5% CO_2_ incubator at 37°C. The isolated EECs were identified by the expression of NPR3 and CDH11 proteins, which were detected using flow cytometry and confocal microscopy.

### RNA transfection and gene silencing

The expression of PLXND1 in the EECs was inhibited by treatment with short hairpin (sh) RNA (GenePharma, Shanghai, China) specific for mice. Three shRNAs targeting *Plxnd1* were cloned into a vector. Cells were transfected with lentivirus vector (LV) carrying a *Plxnd1* RNA system and sh*Plxnd1* or empty control vector using Lipofectamine 3000 (L3000008, Invitrogen, USA) according to the manufacturer’s instructions. After 48 h, the transfection medium was replaced with fresh media and incubated for 24 h, and stable cells were selected using puromycin. The transfection medium was changed 2 days later, and the cells were continuously cultured in fresh media. Western blotting was performed to assess the efficacy of *Plxnd1* silencing or its overexpression in EECs.

### Immunoblotting

For western blotting experiments, EEC lysates were prepared from *in vivo* samples, and immunoblot analyses were performed. EECs were scraped off the plates and lysed in RIPA buffer. Total proteins were detected using the BCA assay (P0012, Beyotime Biotechnology), resolved in running buffer by SDS-PAGE, and transferred onto polyvinylidene fluoride membranes (Millipore) by wet blotting at 100 V for 1 h. Twenty microgram protein samples were mixed with 4X Laemmli buffer (Omp-02, Omiget) and denatured at 95°C for 10 min. Membranes were blocked with 5% nonfat milk and incubated overnight at 4°C with primary antibodies. The antibodies used were microtubule-associated protein 1 light chain three beta (MAP1LC3B; ab192890), PLXND1 (PA5-47012, Invitrogen) and actin beta (ACTB; ab8226, Abcam). Membranes were then incubated with HRP-conjugated secondary antibodies (A0208, Beyotime Biotechnology), visualized by chemiluminescence detection, and quantified using Image QuantTL software (GE Healthcare, Sweden).

### Immunofluorescence

The EECs were fixed in 4% paraformaldehyde (Beyotime Institute of Biotechnology) at room temperature for 10 min. After two PBS washes, the cells were permeabilized with 0.1% Triton 100-X (Beyotime Institute of Biotechnology) at room temperature for 30 min. The EECs were then washed with PBS three times and blocked with blocking buffer (P0260, Beyotime Institute of Biotechnology) at 37°C for 30 min. Samples were then incubated with the primary antibodies diluted in blocking buffer overnight at 4°C, followed by incubation for secondary antibodies for 1 h. The primary antibodies used included anti-MAP1LC3B (ab51520, Abcam), anti-NPR3 (ab97389, Abcam), anti-CDH11 (H00001009, Novus), anti-PLXND1 (PA5-47012, Invitrogen), and anti-ORAI1 (ab244352, Abcam). The secondary antibodies used were FITC-labeled goat anti-rabbit IgG (H + L) (A0423, Beyotime Institute of Biotechnology) and Cy3-labeled goat anti-mouse IgG (H + L) (A0521, Beyotime Institute of Biotechnology). The cells were then digitalized on a Leica SP5 confocal microscope (Leica Microsystems, Germany) and analyzed using Image-Pro Plus 5.0 (Media Cybernetics).

### Infection with GFP-mRFP-LC3 adenoviral vector to monitor autophagy flux.

Primary EECs were infected with an adenoviral vector containing GFP-mRFP-LC3 (LP2100001, HanBio Technology) according to the manufacturer’s instructions as previously described (Yang et al., 2022). After infection for 12 h, the medium was replaced, and the cells were incubated for 24 h. When the infection efficacy reached 70%, the autophagic flux was visualized *via* confocal microscopy (Leica SP5 Leica Microsystems, Germany) by counting the number of RFP and YFP puncta.

### Fluorescence measurements of cytoplasmic Ca^2+^ in intact cells

Measurement of cytoplasmic Ca^2+^ in the EECs was performed using Ca^2+^-sensitive fluorescent Fluo-3-AM (F1241, Invitrogen) as previous described (Yang et al., 2017). The cells that adhered to the glass-bottomed dish were washed with Ca^2+^-free HBSS three times. They were then incubated at room temperature in Ca^2+^-free HBSS containing 5 nM Fluo-3-AM at 37°C for 30 min in the dark. Green fluorescence was observed *via* laser scanning confocal microscopy (LSCM) and imaged for 8 min. Fluorescence intensity (F) was normalized to the baseline fluorescence value F_0_ (F/F_0_) and expressed as [Ca^2+^]_i_. To quantify [Ca^2+^]_i_ in the EECs, we measured the F_max_ and F_min_ of [Ca^2+^]_i_ as previously described. F_max_ was obtained by perfusion with 10 μm ionomycin and 5 mm CaCl_2_; F_min_ was measured by perfusion with 10 mM EGTA and 20 μm BAPTA-AM (B1205, Molecular Probes) in HBSS. The K_d_ of Fluo-3 for Ca^2+^ at room temperature was 400 nm.

### Co-immunoprecipitation

The interaction between PLXND1 and ORAI1 was assessed as previously described (Lin and Lai, 2017). The EEC lysates in the immunoprecipitated (IP) lysis buffer (pH 7.4, 0.025 M Tris, 0.15 M NaCl, 0.001 M EDTA, 5% glycerol) were incubated with antibodies or IgG overnight at 4°C in the dark. The antibodies used for IP included anti-PLXND1 (ab28762, Abcam) and anti-ORIA1 (O8264, Sigma) to immunoprecipitate PLXND1 and ORAI1, respectively. The immunoprecipitated protein complexes were collected by centrifugation at 10,000 × g and washed with an immunoprecipitate elute (26,149, Thermo Scientific Pierce Co-IP kit) to remove unbound immune complexes. The bound immune complex was then analyzed by SDS-PAGE using anti-PLXND1 and anti-ORIA1, respectively.

### Rigid-body docking

To predict the binding affinity of PLXND1 to the ORAI1, we used a rigid body protein–protein docking approach employing docking software: ZDOCK version 3.0.2.(Chen et al., 2003; Mintseris et al., 2007) ZDOCK is a grid-based docking algorithm that uses fast Fourier transforms to accelerate a search in the 6D rotational and translational space, and the three translational degrees of freedom with a 1.2 Å spacing. For each set of rotational angles, only the best-scoring translation is retained, which results in 3,600 or 54,000 predictions for 15° or 6° rotational respectively. The predictions are ranked according to the ZDOCK scoring function, which combines shape complementarity, electrostatics and de-solvation. In the current work, we used the 15 sampling, resulting in a total of 3,600 docking decoys per test case. Higher the Z-score value more excellent will be the binding affinity for protein-protein complex. Each docking setting produces top100 docking results. The conformation with the best docking energy was selected for structural extraction for subsequent research.

### Cell proliferation assays

EEC proliferation activity was tested using a Cell Counting Kit-8 (CCK-8) assay. Cells seeded in 96-well plates were stimulated with various treatments for 24 h. The supernatant was then removed and replaced with 10 μl of CCK-8 solution (C0037, Beyotime Biotechnology) at a density of 10^4^ cells/well for a 2-h incubation period at 37°C. Cell viability was then detected by measuring the absorbance at 450 nm using a microplate reader (Multiskan SkyHigh, Thermo Scientific). Each group was set in five duplicate wells, and the experiments were repeated three times.

### Statistical analysis

All statistical analyses were performed using GraphPad Prism 8 software (version 8.4, San Diego, California) and are shown as mean ± SEM. *t*-tests and one-way analyses were used to evaluate statistical significance. Statistical significance was set at *p < 0.05 and **p < 0.01.

## Results

### Establishment of atrial fibrillation mouse models

**FIGURE 1:**
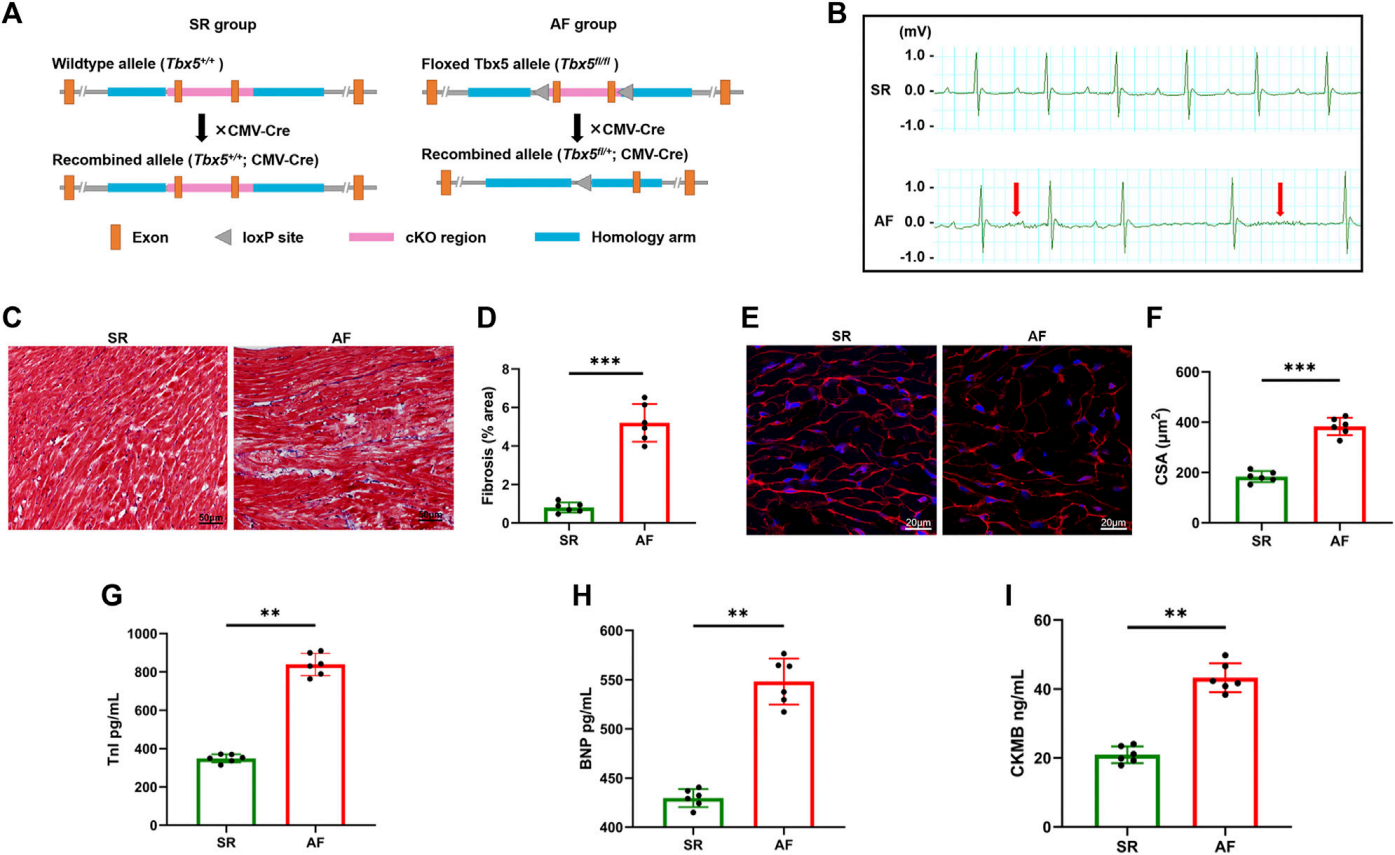
Histological and cellular changes in the hearts of atrial fibrillation mouse models. **(A)**, Experimental schema of AF mice model. Male *Tbx5* ^+/−^ mice (*Tbx5* ^*fl/+*^; CMV-Cre) and CMV-Cre mice were used as SR and AF mice respectively. **(B)** ECG tracing of SR mice (upper) and AF mice (lower); rapid and irregular atrial waves are indicated by red arrows. **(C)** Histological images of hearts from SR and AF mice obtained by Masson’s trichrome staining. Scale bar: 100 μm (right panel). **(D)** The degree of fibrosis was evaluated and calculated by measuring the blue regions (collagen) relative to the total tissue area in sections, as shown in **(C)**. **(E)** Representative images stained with WGA (red) to delineate the sarcolemma, and DAPI (blue). Scale bar, 20 μm. **(F)** Quantification of the CSAs of atrial cardiomyocytes. **(G–I)** Statistics of plasma concentrations of BNP, TnI, and CK-MB in mice in two groups. AF, atrial fibrillation; SR, sinus rhythm; ECG, electrocardiogram; WGA, wheat germ agglutinin; CSA, cross-sectional area; BNP, brain natriuretic peptide; TnI, troponin I; CK-MB, creatine kinase MB.

Floxed mice with deletion of the T-box transcription factor 5 gene (*Tbx5* ^*fl/fl*^) were crossed with CMV-Cre mice to establish the AF mouse model (*Tbx5* ^*fl/+*^; CMV-Cre) (Figure 1A). By electrocardiogram recording, the AF model group showed prolonged AF duration with an irregular heartbeat, which was absent in the control group (Figure 1B), indicating the effective induction of AF. The electrophysiological results showed heterozygous *Tbx5* ^+/−^ mice attenuated AF susceptibility as evidenced by significantly increased AF duration (Supplementary Figure S1). Histological assessment revealed that diffuse myocardial fibrosis (Figures 1C,D) and cardiomyocyte CSA increased (Figures 1E,F) in AF mice compared to that in SR mice. Increased concentrations of serum markers for myocardial injury, including brain natriuretic peptide (BNP), cardiac troponin I (cTnI), and creatine kinase MB (CK-MB), were also observed in the AF group (Figures 1G–I). These data suggest that an increasing AF burden is associated with progressive diffuse cardiac fibrosis and may play a role in adverse cardiac remodeling.

### Atrial fibrillation promotes PLXND1 expression and inhibits autophagy in endocardial endothelial cell

**FIGURE 2:**
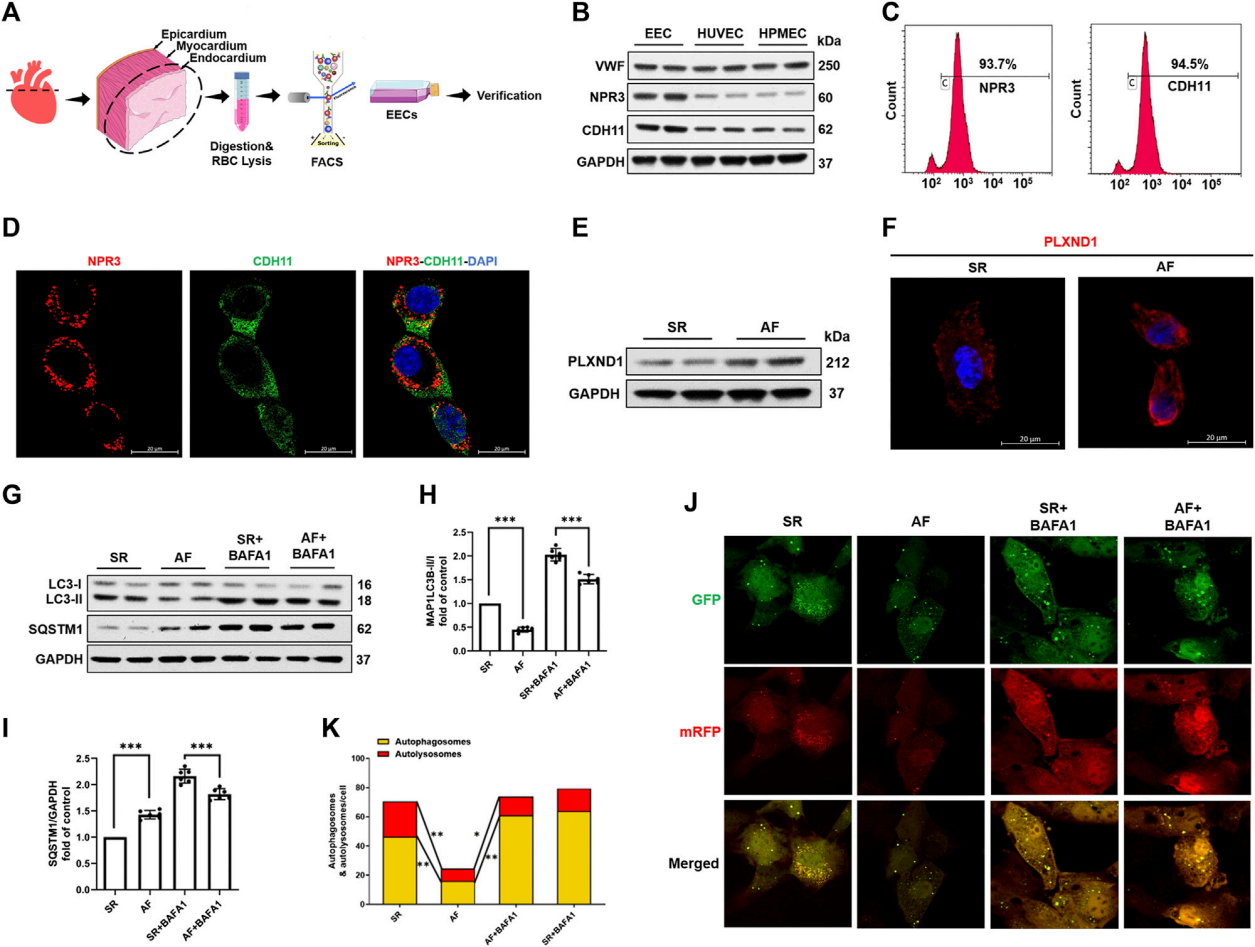
Atrial fibrillation promotes PLXND1 expression and inhibits autophagy in endocardial endothelial cells. **(A)** Diagram of the EEC isolation from SR and AF mice. **(B)** Verification with specific markers (NPR3 and CDH11) in EECs compared to those in HUVECs and HPMECs by WB data. **(C)** Verification with specific markers (NPR3 and CDH11) in EECs compared to those in HUVECs and HPMECs by FACS. **(D)** Immunostaining data verifying the colocalization of EEC marker NPR3 (red) and CDH11 (green) in EECs. **(E)** Representative WB and quantification of PLXND1 in EECs isolated from SR and AF mice. **(G–I)** Representative WB and quantification of autophagy-related protein levels of EECs subjected to BAFA1 (100 nM) treatment or no treatment. **(J)** EECs from SR and AF mice were infected with a tandem GFP-mRFP-LC3 adenovirus for 24 h. Representative images show formation of puncta in different groups. Scale bar: 10 μm. **(K)** Quantitative analysis of yellow and free red puncta in merged images. The number of both yellow and free red puncta decreased in the EECs of AF mice compared to that in SR mice. (*n* = 10 cells per group; cells were isolated from three mice for one experiment and three independent experiments were performed; mean ± SD; *p < 0.05, **p < 0.01). EECs, endocardial endothelial cells; SR, sinus rhythm; AF, atrial fibrillation; HUVECs, human umbilical vein endothelial cells; HPMECs, human pulmonary microvascular endothelial cells; FACS, fluorescence-activated cell sorting; NPR3, natriuretic peptide receptor 3; CDH11, cadherin 11; WB, western blot.

Using classical marker-based sorting, we obtained EECs from SR and AF mice, as described in Figure 2A. The successful isolation of EECs was determined by assessing the presence of specific EC markers, NPR3 and CDH11, in comparison with other human ECs (Figure 2B) and mouse aortic endothelial cells (MAOEC). Flow cytometric analyses of MAOEC (Supplementary Figure S3) showed that 7.63% cells were labeled with NPR3 and 2.06% were labeled with CDH11. More than 88% cells expressed neither NPR3 nor CDH11. As predicted, flow cytometry (Figure 2C) and immunostaining analyses (Figure 2D) revealed that these two markers were robustly enriched in isolated EECs, indicating their high specificity and efficiency for marking EECs. The most significant effect of AF on EECs is the change in the shear force due to blood flow (Schnieder et al., 2019). A recent study revealed that PLXND1 is a novel mechanosensor in ECs and is required for their response to shear stress both *in vitro* and *in vivo* (Mehta et al., 2020). As shown in Figures 2E,F, PLXND1 expression increased in EECs in AF (Figure 2E); PLXND1 was mainly located on the cytomembrane (Figure 2F), where the cells sense shear stress. To confirm the autophagic activity of EECs in AF, we measured the levels of microtubule-associated protein 1 light chain 3b (MAP1LC3B) and SQSTM1/p62, which are general autophagic markers. The western blot analysis showed decreased MAP1LC3B levels (Figures 2G,H) but accumulated SQSTM1 (Figures 2G,I) in the EECs of AF mice. A reduction in LC3-II levels could result from either a decrease in its production due to the inhibition of autophagosome formation or an increase in its degradation. To distinguish between these possibilities, we treated EECs with BAFA1, a known inhibitor of the latter stages of autophagy. Following treatment with BAFA1 (0.1 µm), the decrease in LC3-II levels was no longer detected in EECs from AF mice (Figure 2G), indicating that the decrease in LC3-II in AF mice results from an increase in lysosomal degradation. To further corroborate the aforementioned findings, we used a pH-sensitive tandem GFP-mRFP-LC3 adenoviral construct to monitor the autophagy-induced formation of puncta. Yellow puncta, reflective of the RFP and GFP fluorescence combination, represented autophagosomes, whereas free red puncta (RFP only) represented autolysosomes where acidic pH quenched GFP fluorescence. The number of both free red and yellow puncta (in the merged images) decreased significantly in the EECs of AF mice compared with those in the EECs of SR mice, suggesting a decrease in the number of both autophagosomes and autolysosomes (Figures 2J,K). Interestingly, the effect on autophagy was less obvious between the two groups after treatment with BAFA1 (Figures 2J,K). These data demonstrate that both autophagosome formation and autophagy flux were inhibited in response to AF.

### PLXND1 inhibits autophagy *via* intracellular calcium signaling

**FIGURE 3:**
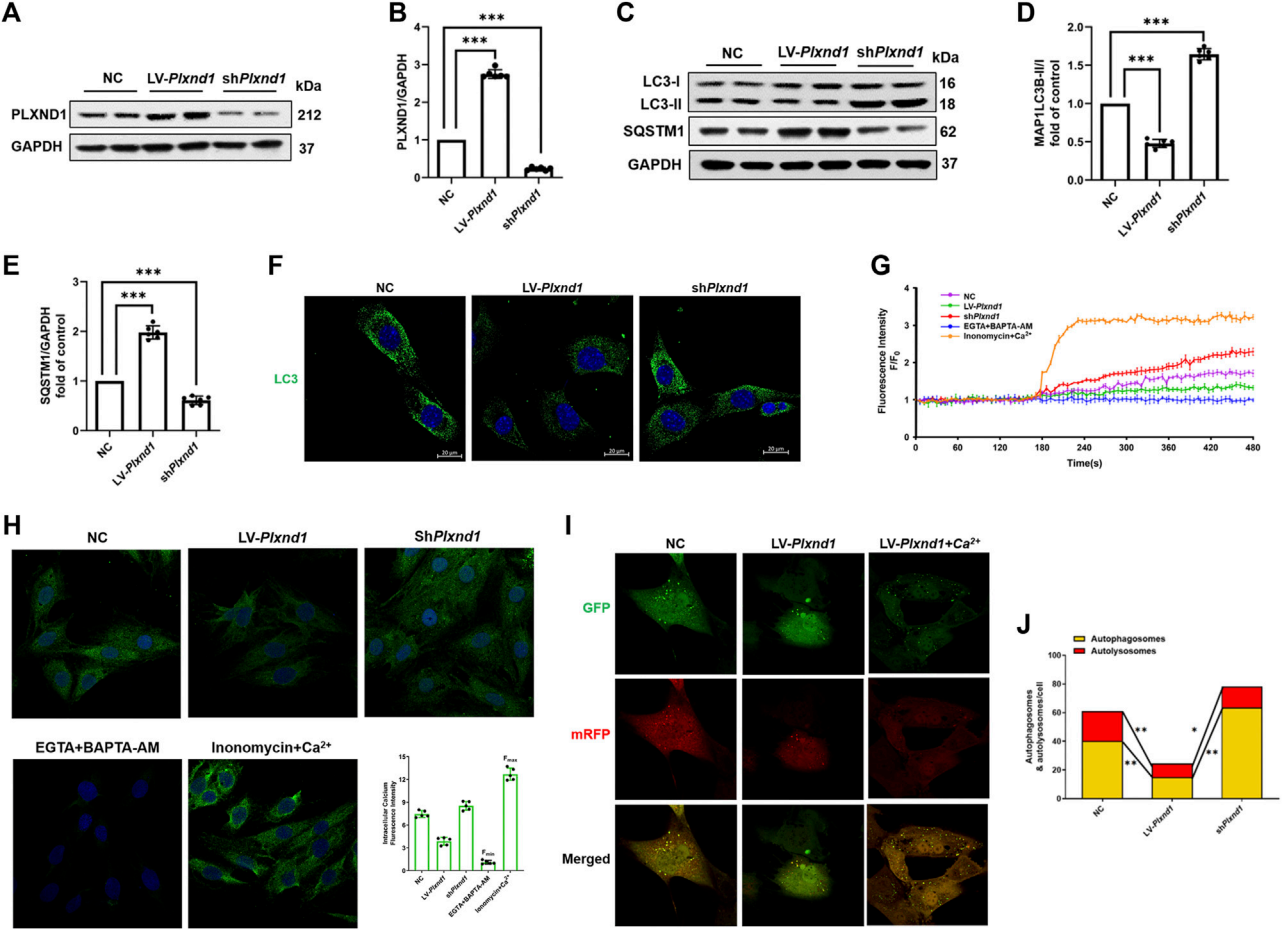
PLXND1 inhibits autophagy via intracellular calcium signaling. Mouse EECs were treated with NC, LV-*Plxnd1*, and sh*Plxnd1*. **(A)** Representative WB of PLXND1 in EECs subjected to the indicated treatments. **(B)** Quantitative analysis of the efficacy of overexpression and knockdown of *Plxnd1*. **(C)** Representative western blots of autophagic markers in EECs subjected to the indicated treatments. **(D–E)** Quantitative analysis indicates that PLXND1 significantly affects the level of autophagy. **(F)** Immunofluorescence staining was used to detect MAP1LC3B in EECs. Scale bar: 20 μm. **(G)** Intracellular calcium was marked by the Fluo-3-AM probe and measured continuously for 8 min **(H)** EECs were incubated with the calcium probe Fluo-3-AM. EGTA + BAPTA-AM was used to obtain the minimum fluorescence intensity (F_min_), whereas ionomycin + calcium was used to obtain the maximum fluorescence intensity (F_max_). Scale bar: 20 μm. Quantitative analysis showed the fluorescence intensity in the different groups. **(I-J)** EECs were infected with a tandem GFP-mRFP-LC3 adenovirus for 24 h. Representative merged images **(I)** and quantitative analysis **(J)** of yellow and free red puncta in merged images of different treatment groups. Scale bar: 10 μm. EECs, endocardial endothelial cells; NC, negative control; PLXND1, plexinD1; WB, western blotting; EGTA, ethylene glycol tetraacetic acid.

Given that the AF-induced inhibition of autophagy was accompanied by an increase in PLXND1 levels, we first overexpressed then knocked-down *Plxnd1* to confirm the association between PLXND1 and autophagy regulation. As shown in Figures 3A,B, the expression of PLXND1 decreased by 90% in cells transfected with sh*Plxnd1* for subsequent experiments. In EECs from SR mice, the overexpression of PLXND1 by lentivirus showed similar autophagy inhibition with decreased LC3B II/I levels (Figures 3C,D), higher SQSTM1 levels (Figures 3C,E), and less fluorescently labeled LC3 (Figure 3F). However, lipid conjugation of free LC3-I to autophagic membrane-associated LC3-II was restored in the extracts of cells with loss of *Plxnd1*. Age-related cardiac disorders, such as heart failure and AF, often present with calcium homeostasis dysfunction. Therefore, intracellular free Ca^2+^ was labeled using Fluo-3-AM. The inhibition of autophagy induced by *Plxnd1* overexpression in EECs was accompanied by a reduction in intracellular Ca^2+^ levels (Figure 3G). Conversely, the knockdown of *Plxnd1* not only affected the autophagic level but also elevated the Ca^2+^ concentration in EECs (Figure 3G). We next attempted to elevate the intracellular Ca^2+^ concentration by supplementing ionomycin with calcium from the culture medium. As illustrated in Figures 3I,J, treatment with LV-*Plxnd1* reduced the number of green fluorescence puncta, and the fluorescence intensity increased significantly in a calcium-rich environment. These results demonstrate that PLXND1 regulates intracellular calcium concentrations by inducing extracellular calcium ion influx into cells and regulating autophagy.

### PLXND1 physically binds with ORAI1 in endocardial endothelial cell to regulate intracellular calcium flux

**FIGURE 4:**
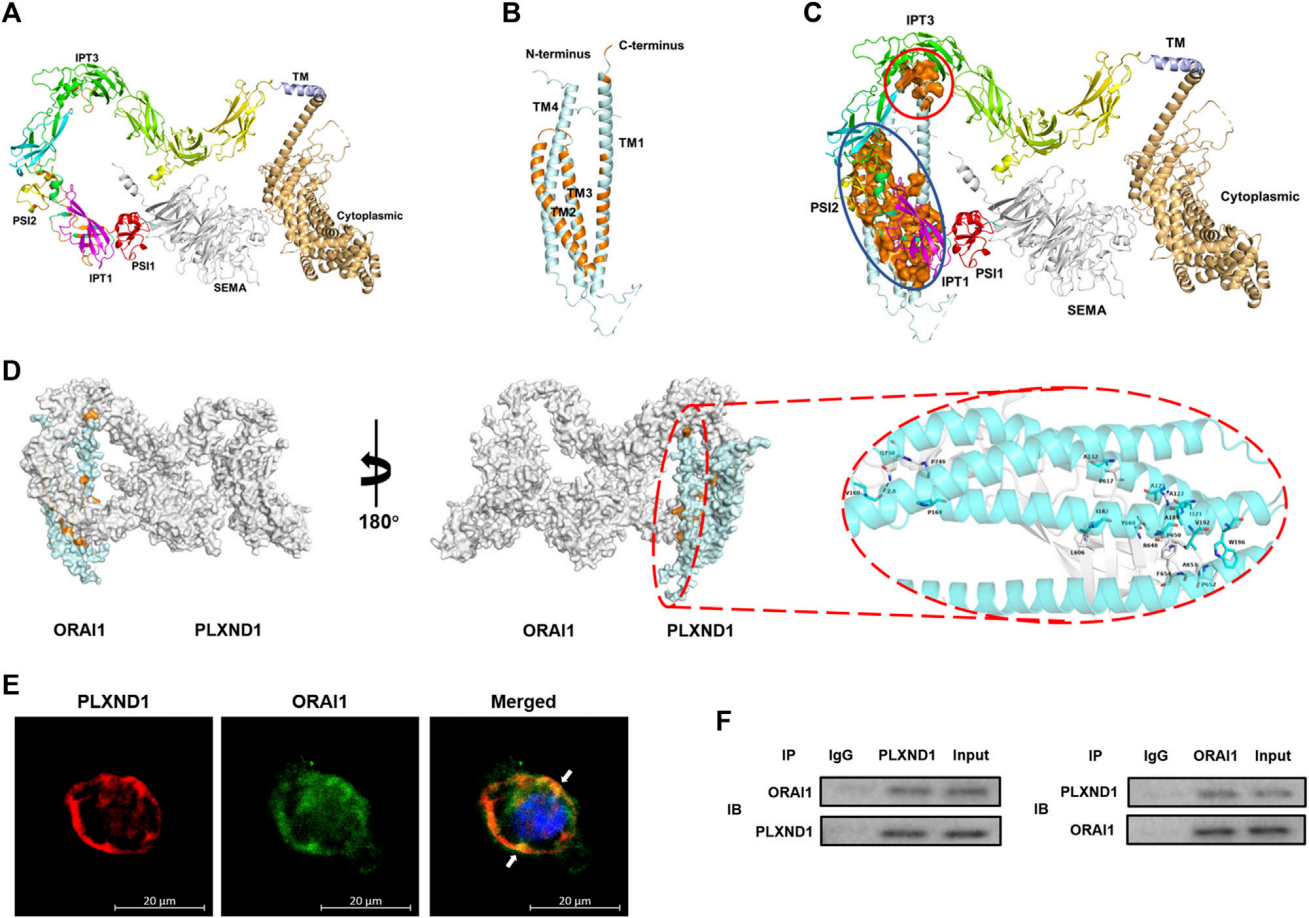
PLXND1 binds to ORAI1 in endocardial endothelial cells to regulate intracellular calcium levels. **(A–B)** Structure of predicted PLXND1 **(A)** and ORAI1**(B)**. The elements of secondary structure (α-helices and β-sheets) are represented by different colors. **(C)** Schematic visualization of the PLXND1-ORAI1 complex, and the coupling regions were marked by red and blue solid circle respectively. **(D)**Three-dimensional structure showing PLXND1-ORAI1 in **(C)**. PLXND1 and ORAI1 are represented as the grey and blue ribbon respectively. A view rotated 180° about the *y* axis is shown with the enlargement of interact surface in red solid circle (right). **(E)** Immunofluorescent staining was applied to mark PLXND1 (red) and ORAI1 (green) in EECs. White arrows represent their colocalization on the EECs. Scale bar: 20 μm. **(F)** Lysates from EECs were subjected to IP with IgG, anti-PLXND1, or anti-ORAI1 antibody, followed by western blotting for PLXND1 and ORAI1. PLXND1, plexinD1; EECs, endocardial endothelial cells; IP, immunoprecipitation; IB, immunoblotting; NC, negative control; WB, western blot.

We further explored how PLXND1 regulates the concentration of intracellular calcium in EECs. Given that ECs are non-excitable cells, and that calcium channels on the membrane mainly pertain to the ORAI and TRPC protein families (Shalygin et al., 2021), we speculated that the insufficient calcium concentration in EECs results from calcium entry dysfunction through ORAI1. Therefore, we investigated whether PLXND1 may have the ability to change the ORAI1, which could potentially alter its control of calcium signals. Then, we conducted protein-protein binding prediction based on the sequence analysis. Structural data showed that PLXND1 has an extracellular SEMA binding domain, trailed by integrin (PSI) domain, immunoglobulin-plexin-transcription (IPT) domain and a cytoplasmic tail containing RasGAP motifs and a GTPase-activating-related domain (Figure 4A). For ORAI1, it contains four transmembrane domains (TM) with both the N-terminus and the C-terminus residing in the cytosol (Figure 4B). We found a physiological binding pattern of PLXND1-ORAI1 (ducking score: 352.15, Figure 4C), which contains more possible binding sites belongs to their functional domains. The overall conformation of the coupling interfaces (orange surface) was identified, one with intracellular region and another with extracellular region (Figure 4D). Specifically, the transmembrane region sequence with amino acids 1812-1815 of PLXND1 assumes a stable conformation binding with the C-terminal portion of ORAI1 (red solid circle, Figure 4C). The later site contains a principal STIM1-activation domain that link ER Ca^2+^ store depletion to the activation of Orai1. In the extracellular binding interface, an ORAI1-TM1 domain (blue solid circle, Figure 4C), the subunit which forms the inner ring of the Ca^2+^ pore naturally (Lunz et al., 2019) also allowed binding of amino acids 620-660 of PLXND1 (Figure 4D). Information on hydrogen bonds and van der Waals contacts in the interface between PLXND1 and ORAI1 were detailed in Supplementary Table S1. Immunofluorescence analysis also indicated that PLXND1 colocalized with ORAI1 on the membrane of EECs (Figure 4E). Finally, we performed co-IP experiments using EEC lysates to assess the interaction between PLXND1 and ORAR1. The results showed that PLXND1 could be immunoprecipitated by ORAI1 *in vitro*, and the negative control IgG did not produce matched bands (Figure 4F). These results demonstrate that PLXND1 physically interacts with ORAI1 on EEC membranes. ORAI1 is a principal component of store-operated calcium channels. These channels are the primary pathways for cellular calcium influx. The modulation of ORAI1 by PLXND1 was based on their interaction and therefore regulated cellular calcium concentrations.

### Decrease in CAMK2 phosphorylation is associated with PLXND1-mediated autophagic inhibition and endocardial endothelial cell dysfunction.

**FIGURE 5:**
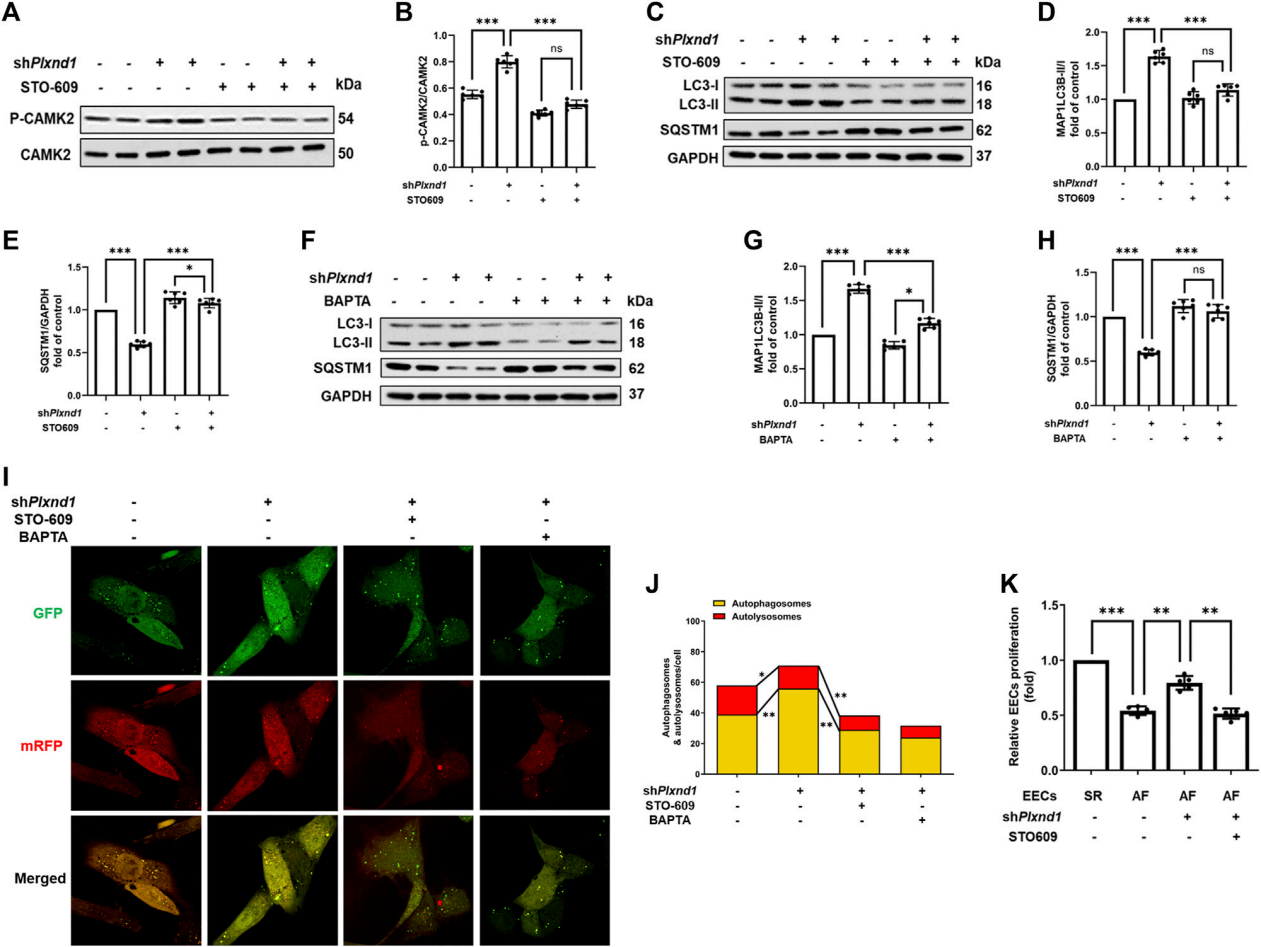
Decrease in CAMK2 phosphorylation is associated with PLXND1-mediated autophagic inhibition and endocardial endothelial cell dysfunction. **(A–B)** Representative WB and quantification of PLXND1-mediated CAMK2 activity in cells subjected to treatment with STO-609 or no treatment. **(C–E)** Representative WB and quantification of PLXND1-mediated autophagic marker levels in cells subjected to treatment with STO-609 or no treatment. **(F–H)** Representative WB and quantification of PLXND1-mediated autophagic marker levels in cells subjected to treatment with BAPTA or no treatment. **(I–J)** EECs were infected with a tandem GFP-mRFP-LC3 adenovirus for 24 h. Representative merged images (I) and quantitative analysis (J) of yellow and free red puncta in merged images in the treated groups. Scale bar: 10 μm. **(K)** EECs from SR and AF mice were seeded in E-plates, and the normalized cell index was recorded in real time for different treatment groups. CAMK2, calmodulin-dependent protein kinase II; PLXND1, plexinD1; WB, western blot; AF, atrial fibrillation; SR; sinus rhythm.

CAMK2 is a well-known effector of cytosolic calcium signaling in various conditions, including autophagy (Høyer-Hansen et al., 2007; Engedal et al., 2013). Given that calcium was largely recovered by *Plxnd1* knockdown, we assessed whether CAMK2 could be activated in mouse EECs. After *Plxnd1* shRNA treatment, CAMK2 activation was strongly increased, as indicated by the elevated levels of p-CAMK2 (Figures 5A,B). As mentioned above, calcium supplementation could prevent the inhibition of autophagy flux caused by the overexpression of *Plnxd1* in EECs. To determine whether the recovery of autophagy is dependent on CAMK2, we pretreated sh*Plxnd1* cells with STO-609, a CAMK2 inhibitor. STO-609 effectively reversed p-CAMK2 levels to basal levels as negative controls, as demonstrated using anti-phospho-CAMK2 (Figures 5A,B). The inhibition of CAMK2 with STO-609 abolished si*Plxnd1*-induced autophagy activation in EECs, as demonstrated *via* western blotting (marked by LC3 and SQSTM1; Figures 5C–E) and immunofluorescence (Figure 5I). A similar decrease in CAMK2 phosphorylation was observed when cells were treated with BAPTA-AM (Figures 5F–H), a membrane-permeable calcium chelator. The autophagic flux was also abolished by blocking intracellular calcium with BAPTA-AM, as represented by a similar decreased number of autophagosomes as that induced by STO-609 (Figures 5I,J). This further confirmed the essential role of CAMK2 in the changes seen in p-CAMK2, SQSTM1, and LC3-II levels. According to the CCK-8 results in Figure 5K, cell proliferation activity was higher in EECs from SR mice than in EECs from AF mice. In the AF mice, PLXND1 shRNA treatment preserved EEC proliferation, and the addition of STO-609 significantly blocked its recovery. The aforementioned inhibition experiments with pharmacological blockers strongly support the hypothesis that the autophagic and proliferative recovery of EECs may partly depend on the activation of CAMK2.

## Discussion

**FIGURE 6:**
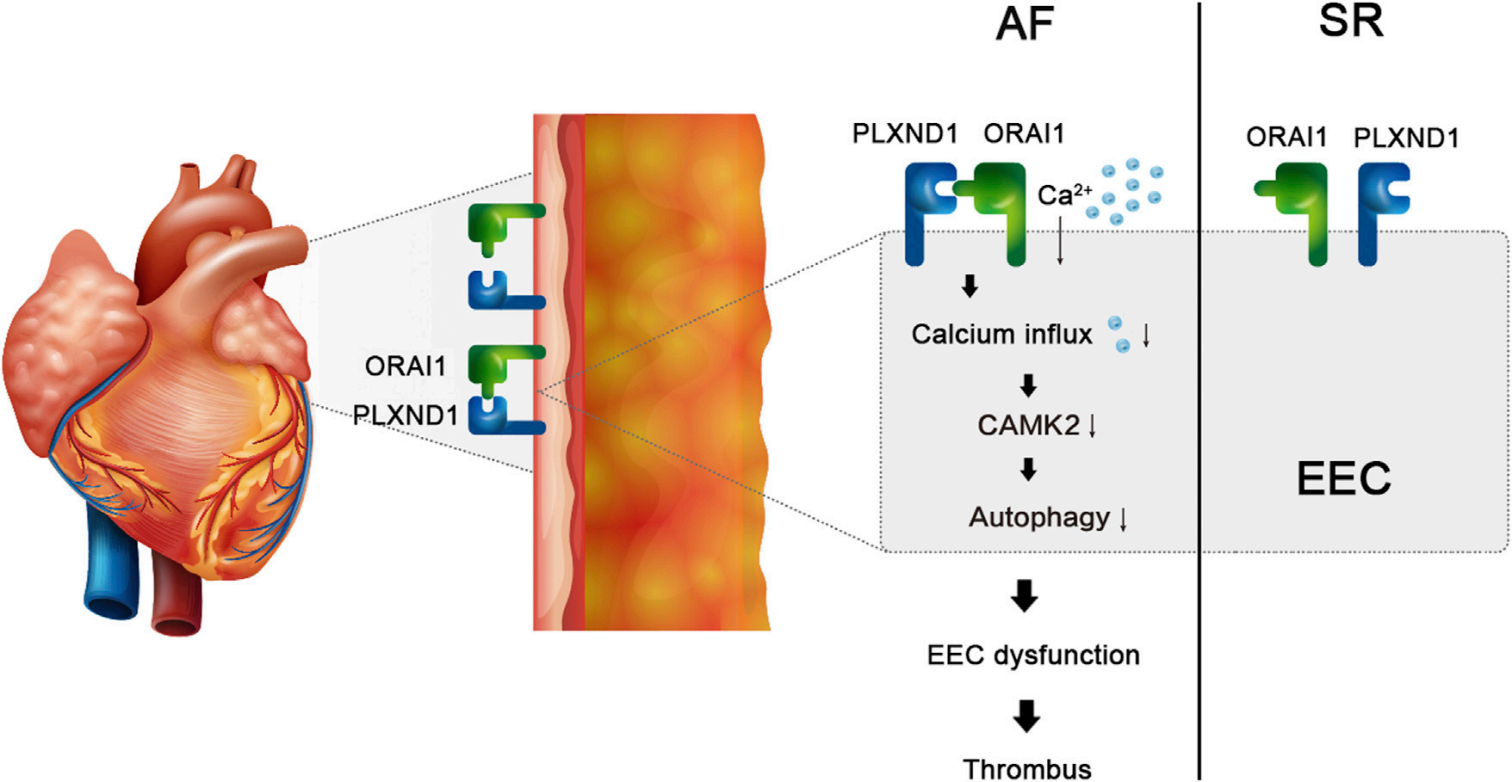
Schematic figure. The expression of the mechanosensitive protein PLXND1 was increased in the cytomembranes of EECs during AF, which served as a scaffold protein to bind with ORAI1 and decreased ORAI1-mediated calcium influx. Decreased calcium influx-mediated phosphorylation of CAMK2 is associated with the inhibition of autophagy and results in EEC dysfunction in AF.

In the present study, we established an AF mouse model and isolated EECs from the endocardium. We showed reduced autophagic flux and intracellular calcium concentrations in EECs from mice with AF. In addition, we detected an increased expression of the mechanosensitive protein PLXND1 in the plasma membrane (PM) of EECs. PLXND1 served as a scaffold protein to bind ORAI1 and decreased ORAI1-mediated calcium influx. Furthermore, we revealed that decreases in the calcium influx-mediated phosphorylation of CAMK1 were associated with autophagy (Figure 6). Our study confirmed, for the first time, that the shear stress-mediated change in PLXND1 expression contributed to intracellular calcium dyshomeostasis, which inhibited autophagic flux in the EECs of AF mice.

AF is the most common type of cardiac arrhythmia worldwide, and the number of patients with AF is predicted to increase steeply in the future (Andrade et al., 2014; Virani et al., 2020). Recent studies have shown that almost 30% of patients with stroke have been previously diagnosed with AF before, during or after the initial event (D'souza et al., 2018). In clinical practice, detachment of the intracardiac thrombus is the primary source of intracranial embolisms (Hart and Halperin, 2001). Most thrombi are located in the LAA as a result of blood flow stagnation and turbulent shear stress changes in AF patients (Aberg, 1969; Desimone et al., 2015). Although vitamin K antagonists and NOACs have been widely used in the clinical treatment of patients with AF to prevent intracardiac thrombus (Steffel et al., 2018), the precise mechanisms leading to thrombosis in the atrium remain elusive. In our study, we focused on the endocardium, which directly faces the bloodstream in the atrium. The inner surface of the endocardium comprises a single layer of ECs, namely, EECs, which express CDH11 and NPR3 (Klein et al., 2020). EEC dysfunction is considered the initial process of thrombosis. Autophagy is the most important mechanism for maintaining cell survival, particularly under adverse conditions (Kaludercic et al., 2020). We detected decreased autophagy flux in EECs from the AF model; therefore, we considered that autophagy flux inhibition may contribute to the AF-mediated dysfunction of EECs.

Autophagy is a highly conserved cellular self-digestion pathway that recycles amino acids and other degradation products in the cytoplasm and helps maintain cellular homeostasis (Dikic and Elazar, 2018). Moderate autophagy exerts a cardioprotective effect, which may be either more or less deleterious in the heart (Yang et al., 2017; Kaludercic et al., 2020). Previous studies have shown that patients develop postoperative AF as a result of impaired cardiac autophagy after CABG (Garcia et al., 2012). Another study indicated that canonical AMPK- and Akt-mTOR-mediated autophagy had a myocardial protective effect during ischemia and reperfusion (Paiva et al., 2011). In contrast, recent reports revealed that autophagic flux was markedly activated in the atria of patients with persistent AF and in a rabbit model of atrial rapid pacing. They further provided evidence that autophagy induces atrial electrical remodeling *via* the ubiquitin-dependent selective degradation of L-type calcium channels (Cav1.2) (Yuan et al., 2018). Therefore, the level of autophagic flux may depend on the disease and cell type, even in the heart. In the present study, we found decreased autophagic flux in EECs in the AF model and a decrease in the intracellular calcium concentration. This confirmed that calcium signaling is associated with autophagy inhibition.

A growing body of evidence has suggested that abnormal intracellular calcium handling may play a role in both the initiation of AF episodes and cellular remodeling processes (Dobrev and Wehrens, 2017; Dridi et al., 2020; Nattel et al., 2020). Most studies have focused on the RyR2 calcium channel from which spontaneous sarcoplasmic reticulum calcium release activates the Na^+^/Ca^2+^-exchanger (NCX) in cardiomyocytes (Dridi et al., 2020). This triggers the action potential, leading to focal ectopic firing. In contrast, EECs serve as non-excited cells, and the ORAI and TRPC families are considered to be the major calcium entry pathways (Shalygin et al., 2021). In the present study, we utilized a calcium fluorescence probe to mark calcium ions in EECs and found that ORAI1-mediated calcium influx was decreased. ORAI1 is a key component in the Ca^2+^ release-activated Ca^2+^ (CRAC) channel complex located in the plasma membrane (Prakriya et al., 2006). It is commonly accepted that ORAI1 directly binds to STIM1 in the ER to trigger calcium influx (Prakriya et al., 2006; Hogan, 2015). In addition to STIM-ORAI1 signaling or STIM1-ORAI1-TRPC1 signaling that can regulating store-operated calcium entry (SOCE) (Ambudkar et al., 2017). Our results suggest a different model where ORAI1 C- terminus and TM1 allows binding of another PM protein, PLXND1. We highlight the specific role of PLXND1 in gating of the ORAI1 channel by influencing their conformation, influencing the opening of ORAI1 channels as well as the selectivity filter of the ORAI1. Conversely, PLXND1 may link to ORAI1 subunits and block their assembly as tetramers and hexamers in the PM. However, understanding the precise mechanism by which PLXND1 decreases ORAI1-mediated calcium influx requires further investigation.

PLXND1 is a cellular receptor with functions that have mostly been explored in different tumors (Roodink et al., 2005; Casazza et al., 2010; Jurcak et al., 2019). Recently, PLXND1 was found to serve as a mechanosensor for ECs (Mehta et al., 2020). Under laminar flow conditions, PLXND1 maintained the alignment of ECs in the flow direction. Once the bloodstream was disturbed, PLXND1 expression was upregulated, which promoted pro-inflammatory processes in atherosclerosis. The biological role of PLXND1 in the detection of mechanical force was first verified in mouse lung ECs and bovine aortic endothelial cells (BAECs). Considering the hallmark, disturbed blood flow, during AF (Fatkin et al., 1994), we proposed a new hypothesis for the force sensor function of PLXND1 in EECs that cover the inner surface of the cardiac chamber rather than the arterial lumen. PLXND1 was found to be abundantly expressed in EECs and showed significantly elevated levels in the AF models compared with those in mice with SR. Furthermore, we showed that PLXND1 converts mechanical force into a cellular biological signal, namely intracellular calcium signal, and further regulates autophagy and EEC function. The dysfunction of EECs is a necessary condition for intracardiac thrombogenesis (Watson et al., 2009), which is a potential risk factor for stroke and other arterial embolisms in AF patients. Therefore, PLXND1-mediated autophagy regulation and the development of a monoclonal antibody targeting PLXND1 in EECs may be a potential mechanism of antithrombotic therapy for AF. We will conduct further *in vivo* experiments centering on the aforementioned hypotheses.

## Data Availability

The original contributions presented in the study are included in the article/Supplementary Material, further inquiries can be directed to the corresponding authors.
