# Supplementary material for: PLXND1-mediated calcium dyshomeostasis impairs endocardial endothelial autophagy in atrial fibrillation
Source: Front Physiol. 2022 Aug 9;13:960480. doi: 10.3389/fphys.2022.960480 (PMC9395636; doi:10.3389/fphys.2022.960480)
Supplement: Supplementary file 1 [file Table1.DOCX]

**Supplementary Table S1. The interaction pair of the best-scoring PLXND1-ORAI1 complex**

| Interaction Pair | Distance (Å) | Category | Types |
| --- | --- | --- | --- |
| PLXND1-ORAI1 |  |  |  |
| A:GLN750:NE2 - B:VAL160:O | 2.76 | Hydrogen Bond | Conventional Hydrogen Bond |
| A:PHE654 - B:ALA189 | 3.26 | Hydrophobic | Pi-Alkyl |
| A:PRO617 - B:ALA132 | 3.28 | Hydrophobic | Alkyl |
| A:LEU606 - B:ILE182 | 3.87 | Hydrophobic | Alkyl |
| A:ALA653 - B:TRP196 | 4.00 | Hydrophobic | Pi-Alkyl |
| A:PRO650 - B:ALA122 | 4.21 | Hydrophobic | Alkyl |
| A:PRO749 - B:PRO164 | 4.24 | Hydrophobic | Alkyl |
| A:ALA653 - B:VAL192 | 4.26 | Hydrophobic | Alkyl |
| A:ARG648 - B:ILE121 | 4.36 | Hydrophobic | Alkyl |
| A:GLU840:OE1 - B:LYS161:NZ | 4.44 | Electrostatic | Attractive Charge |
| A:PRO652 - B:TRP196 | 4.50 | Hydrophobic | Pi-Alkyl |
| A:TYR660 - B:ALA125 | 4.65 | Hydrophobic | Pi-Alkyl |
| A:ILE620 - B:LEU186 | 4.66 | Hydrophobic | Alkyl |
| A:PHE654 - B:VAL192 | 4.69 | Hydrophobic | Pi-Alkyl |
| A:PRO723 - B:ILE172 | 4.80 | Hydrophobic | Alkyl |
| A:PHE725 - B:CYS143 | 4.84 | Hydrophobic | Pi-Alkyl |
| A:PRO617 - B:VAL129 | 5.00 | Hydrophobic | Alkyl |
| A:ILE620 - B:VAL129 | 5.00 | Hydrophobic | Alkyl |
| A:TYR660 - B:CYS126 | 5.16 | Hydrophobic | Pi-Alkyl |
| A:GLU609:OE1 - B:LYS265:NZ | 5.18 | Electrostatic | Attractive Charge |
| A:TYR660 - B:VAL129 | 5.43 | Hydrophobic | Pi-Alkyl |
| A:PHE654 - B:LEU193 | 5.44 | Hydrophobic | Pi-Alkyl |
