# Supplementary material for: PLXND1-mediated calcium dyshomeostasis impairs endocardial endothelial autophagy in atrial fibrillation
Source: Front Physiol. 2022 Aug 9;13:960480. doi: 10.3389/fphys.2022.960480 (PMC9395636; doi:10.3389/fphys.2022.960480)
Supplement: Supplementary file 2 [file DataSheet2.docx]

**Supplementary Figures and Legends**


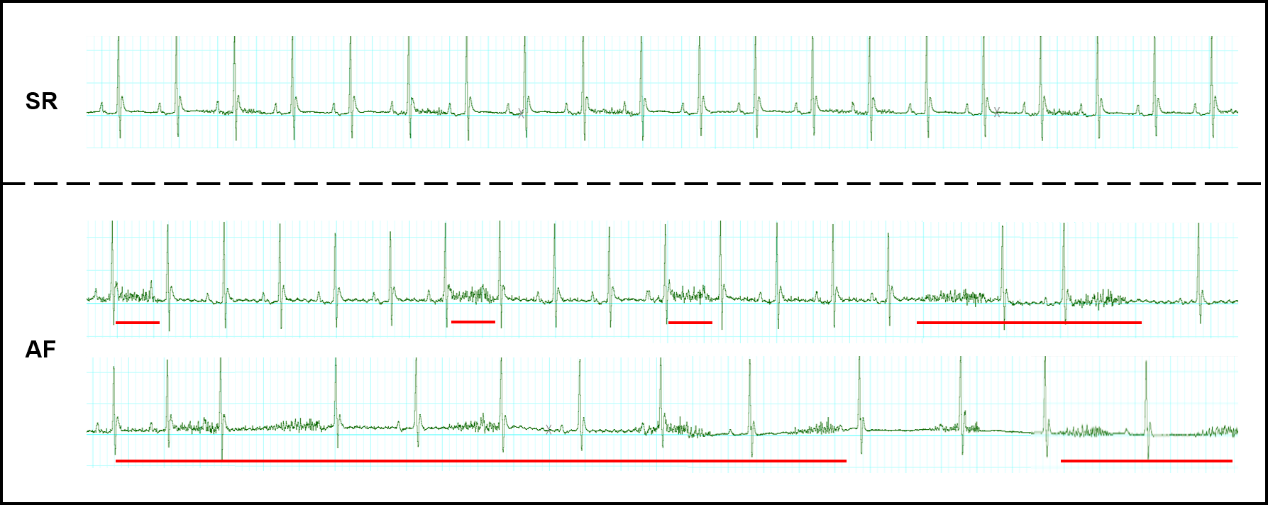


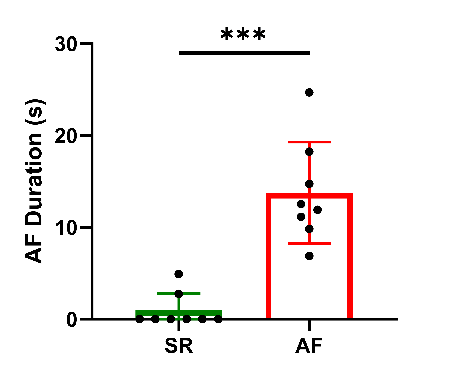


**Supplementary Figure 1.** Representative ECGs and bar graphs showed mice with increased AF duration in AF group (n = 8) compared with SR group (n = 8).


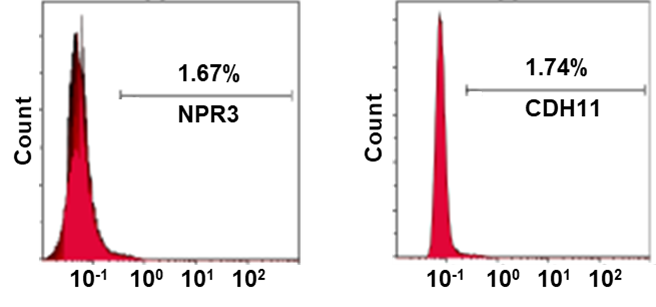


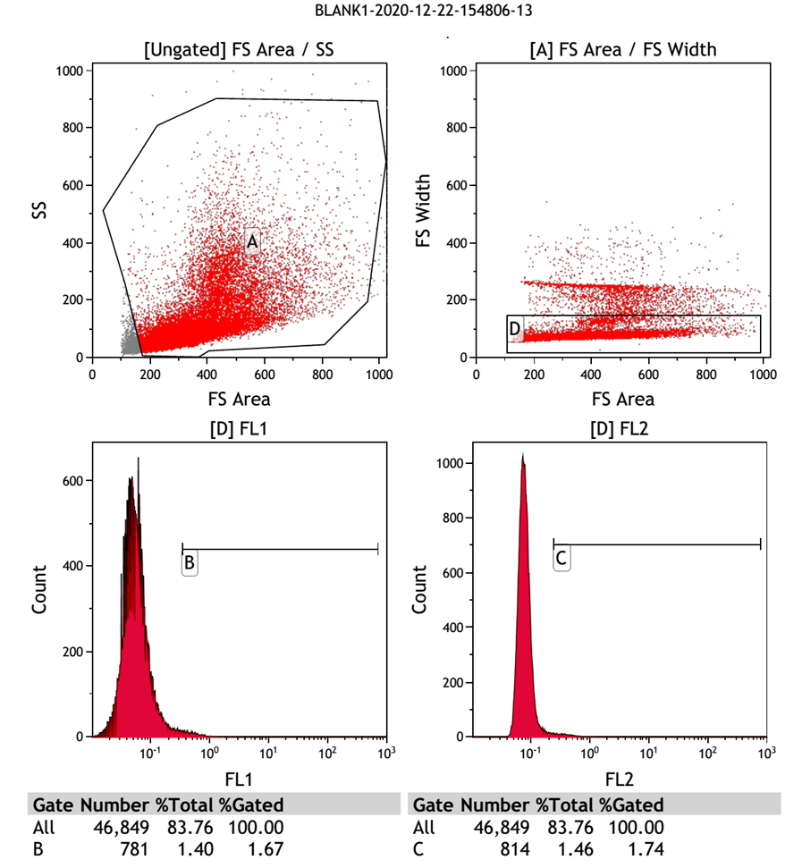


**Supplementary Figure 2.** The negative controls and corresponding gating strategy for sorting EECs (NPR3+ and CDH11+) based on different scatter properties.


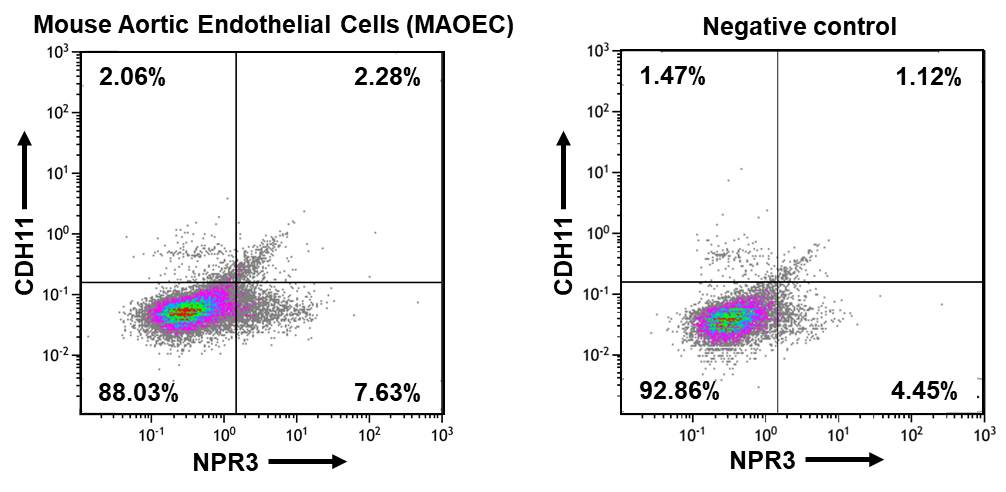


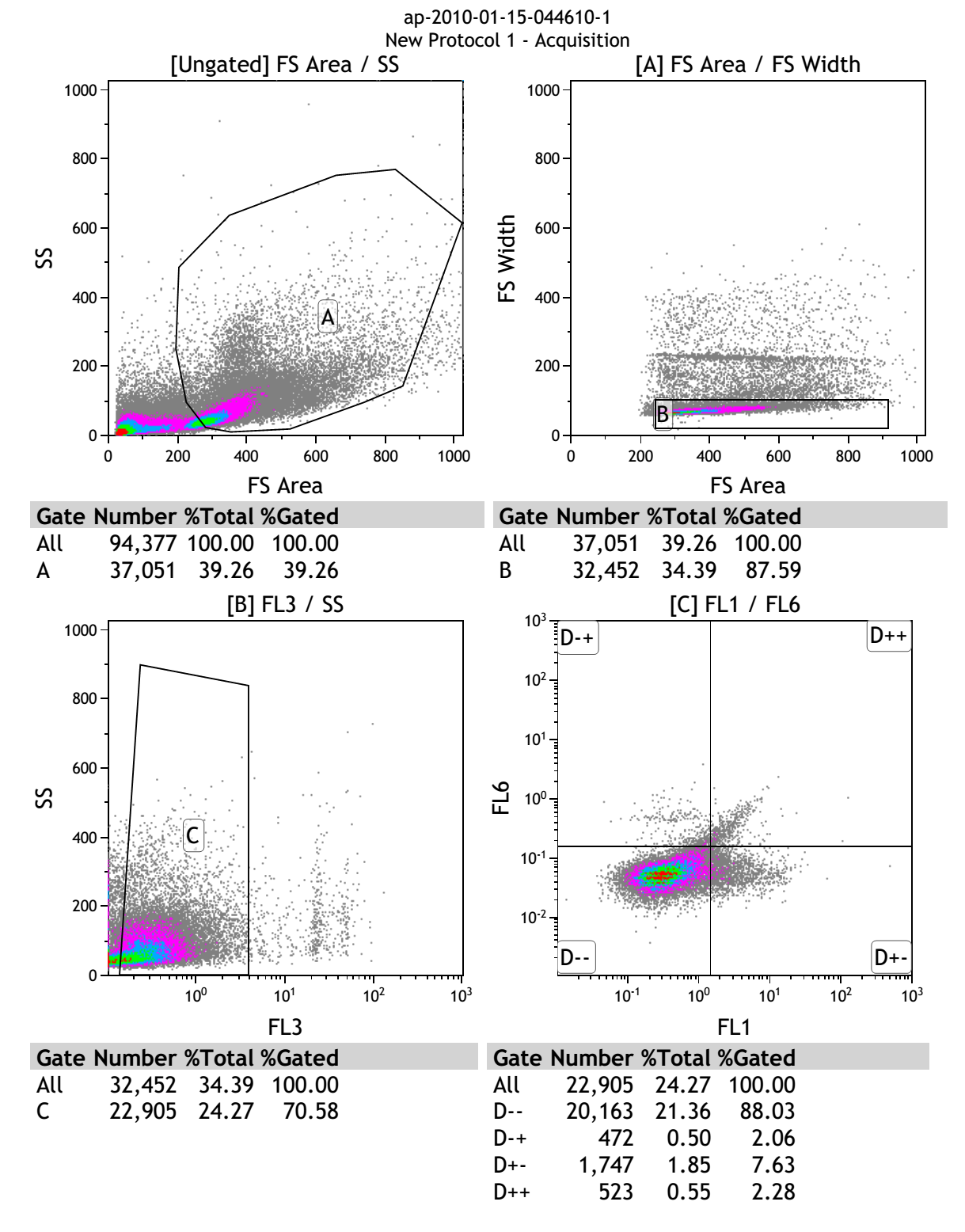

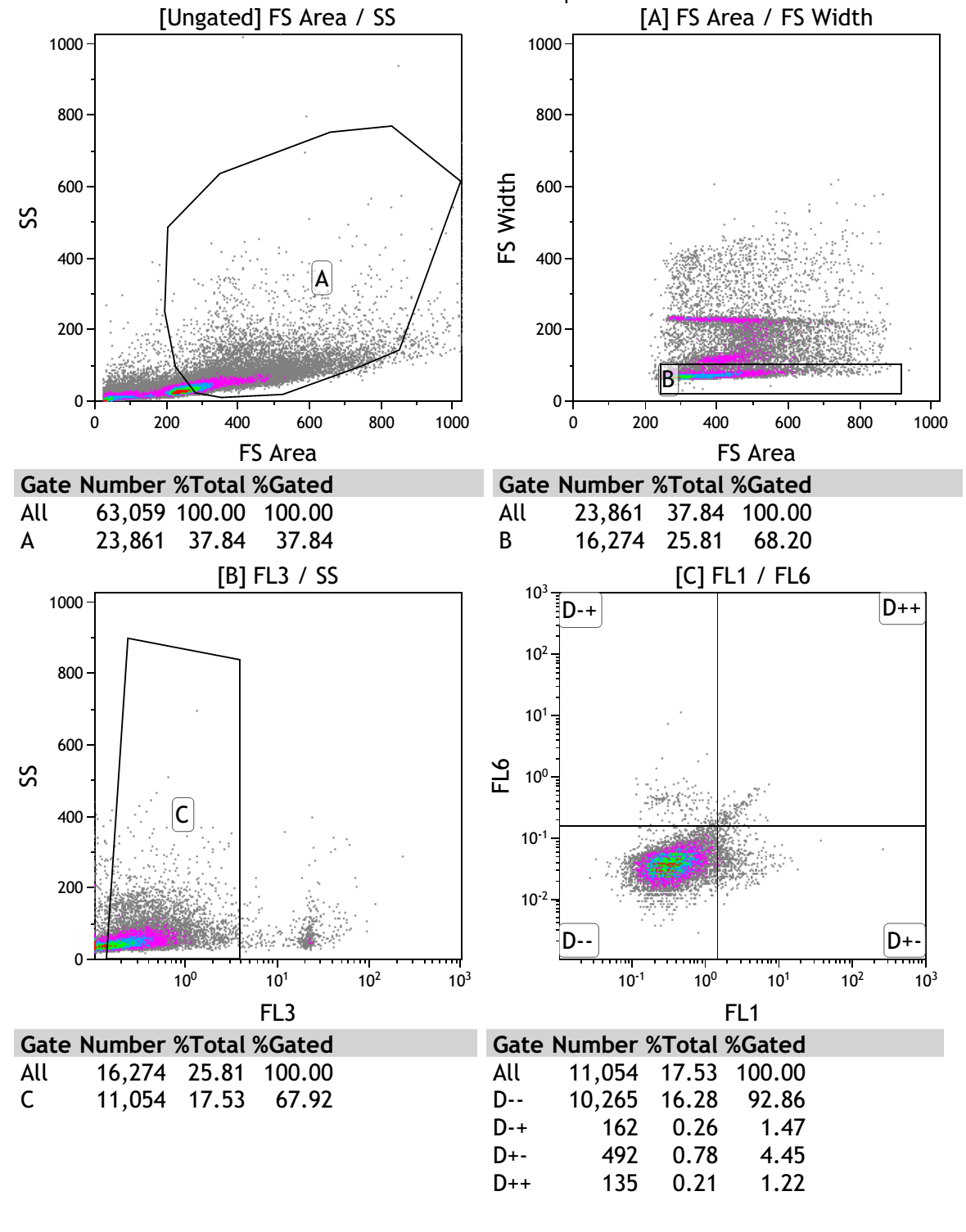


**Supplementary Figure 3.** Flow cytometric analysis of NPR3 and CDH11 expression on mouse aortic endothelial cells (MAOEC).
